# Polyphenolic Extract from *Sambucus ebulus* L. Leaves Free and Loaded into Lipid Vesicles

**DOI:** 10.3390/nano10010056

**Published:** 2019-12-25

**Authors:** Ramona-Daniela Păvăloiu, Fawzia Sha’at, Corina Bubueanu, Mihaela Deaconu, Georgeta Neagu, Mousa Sha’at, Mihai Anastasescu, Mona Mihailescu, Cristian Matei, Gheorghe Nechifor, Daniela Berger

**Affiliations:** 1 National Institute for Chemical-Pharmaceutical Research and Development-ICCF Bucharest, Vitan Avenue 112, 031299 Bucharest, Romania.; fawzya.shaat@gmail.com (F.S.); corina.bubueanu@yahoo.com (C.B.); getabios@yahoo.com (G.N.); 2 Faculty of Applied Chemistry and Materials Science, University “Politehnica” of Bucharest, 1–7 Gheorghe Polizu St., 011061 Bucharest, Romania; mihaela_deaconu@yahoo.com (M.D.); cristian.matei@upb.ro (C.M.); doru.nechifor@yahoo.com (G.N.); 3 Faculty of Pharmacy, University of Medicine and Pharmacy Grigore T. Popa, Iasi, Universitatii Avenue, 16, 700115 Iasi, Romania; mousa.shaat1@gmail.com; 4 “Ilie Murgulescu” Institute of Physical-Chemistry, Romanian Academy, Splaiul Independentei no. 202, 060021 Bucharest, Romania; manastasescu@icf.ro; 5 Physics Department, University “Politehnica” of Bucharest, Splaiul Independetei no 313, 060042 Bucharest, Romania; mona.mihailescu@yahoo.com

**Keywords:** *Sambucus ebulus*, lipid vesicles, polyphenols, cytoprotective effect

## Abstract

The paper deals with the preparation and characterisation of hydroalcoholic polyphenolic extract from *Sambucus ebulus* (SE) leaves that was further loaded into three-types of lipid vesicles: liposomes, transfersomes, and ethosomes, to improve its bioavailability and achieve an optimum pharmacological effect. For *Sambucus ebulus* L.-loaded lipid vesicles, the entrapment efficiency, particle size, polydispersity index and stability were determined. All prepared lipid vesicles showed a good entrapment efficiency, in the range of 75–85%, nanometric size, low polydispersity indexes, and good stability over three months at 4 °C. The in vitro polyphenols released from lipid vehicles demonstrated slower kinetics when compared to the free extract dissolution in phosphate buffer solution at pH 7.4. Either free SE extract or SE extract loaded into lipid vesicles demonstrated a cytoprotective effect, even at low concentration, 5 ug/mL, against hydrogen peroxide-induced toxicity on L-929 mouse fibroblasts’ cell lines. However, the cytoprotective effect depended on the time of the cells pre-treatment with SE extract before exposure to a hydrogen peroxide solution of 50 mM concentration, requiring at least 12 h of pre-treatment with polyphenols with radical scavenging capacity.

## 1. Introduction

*Sambucus ebulus* L. (fam. Adoxaceae) (SE) is a herbaceous, perennial plant, commonly known as dwarf elder or danewort, and is widespread from Central Europe to the Middle East. Generally, SE leaves are widely used in herbal medicine for the treatment of kidney and lung diseases, inflammation-related gastrointestinal disorders, rheumatoid arthritis, fever, infections, sore throat, and bites [1,2,3,4].

Several studies revealed that SE leaves comprise various compounds such as flavonoids, polyphenols, triterpenes, tannins, iridoids, phytosterols, lectins, sugars, fibers, vitamins, and minerals [5,6,7,8]. The main therapeutic benefits of SE extracts are related to their antioxidant and anti-inflammatory properties, making them valuable sources for food, cosmetic and pharmaceutical industries, as well as medicine. However, the compounds from SE extract exhibit poor water solubility, which limits their bioavailability and therapeutic applications. Also, because of domestic processes (thermal and mechanical) and storage conditions, most of the bioactive substances of SE extract are susceptible to degradation, and thus, a significant portion of phytochemicals is not absorbed [9,10].

A solution to overcome these drawbacks could be the encapsulation of SE extract in various carriers. For the encapsulation of bioactive compounds or extracts, several carriers have been reported: *(i)* natural or synthetic polymers [11,12]; *(ii)* micro/nanofibers [13,14]; *(iii)* lipids including micro/nanoemulsions, lipid vesicles and solid lipid nanoparticles [15,16,17,18]; *(iv)* cyclodextrins [19,20]; and *(v)* other types, e.g., niosomes, inorganic nanoparticles and nanocrystals [21,22].

The encapsulation into lipid vesicles causes better stability, less impact on natural compounds, decreased toxicity and side effects, and lower frequency of dose administration, and thus, enhanced patient compliance and therapeutic efficiency [23,24]. Successful attempts to enclose bioactive compounds into lipid vesicles, like liposomes, transfersomes, ethosomes, etc., are reported [25,26]. Liposomes are microscopic spheres with an aqueous core surrounded by one or more outer shells consisting of lipids in a bilayer [27], while transfersomes are elastic or flexible liposomes which consist of lipids and a single chain surfactant (e.g., sodium cholate, deoxycholate, Span 80, Tween 80) that acts as an “edge activator” and destabilizes the lipid bilayers, providing greater flexibility comparing to the liposomes [28]. Novel lipid vesicles called ethosomes are other possible carriers which consist of a lipid layer and a high ethanol concentration (20–40%), providing greater flexibility than that of liposomes [29].

Only a few studies on the encapsulation of bioactive compounds from *Sambucus* species were reported. For instance, Stanciuc et al. [30] encapsulated bioactive compounds from *Sambucus nigra* L in whey proteins and a combination of whey proteins isolates and pectin proving higher heat stability. Sobieralska and Kurek [31] demonstrated the superior benefits of *Sambucus nigra* extract encapsulated in maltodextrin-β-glucan microcapsules in comparison with maltodextrin with Arabic gum ones in terms of efficiency and stability. Moreover, Bryła et al. [32] compared three types of lecithins (egg yolk, soybean and sunflower) for obtaining liposomes loaded with *Sambucus nigra* L extract, and none of lecithins produced structures with good stability and encapsulation efficiency simultaneously.

Herein, we report the design of lipid vesicles for encapsulation SE leaves extract for improved efficiency. To our knowledge, this is the first report regarding the encapsulation of SE leaves extract in three types of lipid vesicles, including liposomes, transfersomes, and ethosomes. Furthermore, the radical scavenger activity of SE leaves extract free and loaded on lipid vesicles on the L-929 mouse fibroblasts cell line was assessed, resulting in a cytoprotective effect of cells.

## 2. Materials and Methods

### 2.1. Materials

Phosphatidylcholine from egg yolk (PC), cholesterol, sodium cholate, Triton X-100, Folin-Ciocalteu reagent, 2,2-diphenyl-1-picrylhydrazyl (DPPH), and sodium carbonate were purchased from Sigma-Aldrich Co. (Merck Group, Darmstadt, Germany). For chromatographic analysis, the following standard HPLC-grade compounds were used: Gallic acid (Alfa Aesar, Ward Hill, MA, USA 98%), protocatechuic acid (Tokyo Chemical Industry, Tokyo, TCI, Japan >98%), catechin hydrate (Sigma, Merck Group, Darmstadt, Germany >98%), vanilic acid (TCI, >98%, GC-grade), caffeic acid (Sigma, 98%), syringic acid (Molekula GmbH, Munich, Germany, >98.5%), (-)epicatechin (TCI, >98%), quercetin (Sigma, >95%, HPLC-grade), rutin hydrate (Sigma, 95%), chlorogenic acid (HWI Group, Alpen Aan de Rijn, The Netherlands, primary reference standard), *trans*-*p*-coumaric acid (Sigma-Aldrich, analytical standard), myricetin (Sigma, >96%), rosmarinic acid (Sigma, >98%), *trans-*resveratrol (Sigma-Aldrich, certified reference material), kaempferol (Sigma, >97%). The solvents used for mobile phases and samples preparation were acetonitrile (Riedel-de Haen, Honeywell Riedel-de-Haën, Seelzer, Germany), ethanol (Riedel-de Haen), formic acid (Merck Group, Darmstadt, Germany), and ultrapure water (Millipore Direct-Q3UV water system, version Q3UV, catalog product no. C9185, Merck Group, Darmstadt, Germany) equipped with a Biopack UF cartridge. For oxidative stress induction assay we used Eagle’s Minimum Essential Medium (EMEM), Phosphate-Buffered Saline (PBS), fetal equine serum (FES), 0.25% Trypsin-Ethylene-Diamine-Tetraacetic Acid (EDTA) solution, penicillin-streptomycin-neomycin mixture in 0.9% NaCl (10,000 µg/mL/10,000 U/mL) (PSN), L-ascorbic acid powder, γ-irradiated, hydrogen peroxide aqueous solution 30% (*w*/*w*) purchased from Sigma-Aldrich Co. (Germany). We used L-929, murine fibroblast cell line (ATCC^®^ CRL-6364™), cultured in EMEM, high-glucose medium supplemented with 10% FES and 1% PSN, at 37 °C in an atmosphere of 5% CO_2_. The *Cell Titer 96^®^ A Queous Non-Radioactive Cell Proliferation Assay* was supplied from Promega Corporation (Madison, WI, USA).

### 2.2. Vegetal Material

The SE leaves were harvested at their maturity from Dambovita County, Romania (latitude: 45°18′15.4′′ N, longitude: 25°23′28.4′′ E) and identified by the botanical team of INCDCF-ICCF Bucharest, Romania. SE leaves were selected as vegetal material for the extract preparation considering their bioactive phytochemicals, including polyphenolic compounds, which determined protection against reactive oxygen species [33].

### 2.3. Preparation of SE Extract

The SE leaves were washed with distilled water, dried at room temperature (25 °C), and milled into a fine powder. The grounded vegetal material (50 g) was put in 500 mL 50% (*v*/*v*) ethanol-water mixture, and then refluxed for 1 h under stirring. The resulting suspension was filtered off and evaporated at reduced pressure (72–75 mm Hg), at 60 °C, using a rotary evaporator (Laboranta 4000, Heidolph Instruments GmbH & Co. KG, Schwabach, Germany) and then re-dissolved in 50% ethanol aqueous solution. The obtained SE extract with 47.20 mg/mL concentration, whose extraction yield was 23.6%, was stored in a refrigerator at 4 °C.

### 2.4. Determination of Total Polyphenols Content

The total polyphenol content of SE extract was determined using the Folin-Ciocalteu method [34]. Briefly, 1 mL extract, 10 mL of water and 1 mL of Folin-Ciocalteu reagent (diluted 10 times) were mixed, and the volume was completed up to 25 mL with 5% (wt) aqueous solution of sodium carbonate. After 30 min, the absorbance of the resulted solution was read at 760 nm, using a UV/VIS spectrophotometer (Helios λ, ThermoFisher Scientific, Waltham, MA, USA). The total polyphenolic compounds content was determined based on a calibration curve for gallic acid (0.01–0.1 mg/mL concentration range; *y* = 0.01322*x* + 0.0272, *R*^2^ = 0.99574). The results were presented as milligrams of gallic acid equivalents per gram of dry extract (mg GAE/g).

### 2.5. High-Performance Liquid Chromatography Analysis of SE Extract

The phenolic compounds separation, identification and quantification were performed using reverse-phase high-performance liquid chromatography (HPLC; Shimadzu Nexera 2, Shimadzu Corporation, Kyoto, Japan) with a photodiode array detector (SPD-M30A) operating in the 250–650 nm wavelength range and a LC-20ADXR quaternary pump (Shimadzu Corporation), DGU-20A_5R_ vacuum degasser (Shimadzu Corporation), SIL-30 AC autosampler (Shimadzu Corporation) and CTO-20AC column oven (Shimadzu Corporation). The chromatograms were acquired and processed with LabSolutions Lite LC/GC software (version 5.82, Shimadzu Corporation). The separation was performed by gradient elution on a Nucleosil^®^ reversed-phase C_18_ column (Macherey-Nagel GmbH & Co. KG, Düren, Germany) 4.6 × 100 mm (2.7 µm) using two mobile phases: 2.5% aqueous formic acid solution (mobile phase A) and 90% aqueous acetonitrile with 2.5% formic acid (mobile phase B). The elution program was established after modification of the method proposed by Nicoletti et al. [35]: 1.5 min isocratic elution with 5% mobile phase B, followed by 6 min linear gradient to 9% mobile phase B and another 6 min linear gradient to 13.5% B, isocratic elution with 13.5% B for 2.5 min, 5 min linear gradient to 18.5% B, followed by 1 min linear gradient to 22.5% B, isocratic elution with 22.5% mobile phase B for 3.5 min, 4.5 min linear gradient to 35% mobile phase B, 5 min linear gradient to 100% mobile phase B and isocratic elution for 5 min with 100% mobile phase B. At the end, the gradient elution was returned to the initial concentration, and before the next injection the column was equilibrated for 10 min. The flow rate was set at 0.4 mL/min, and the separation was performed at 20 °C, using an injection volume of 1 µL. All solvents used for the mobile phase were previously filtered off through 0.45 µm nylon membranes and degassed. The chromatogram of SE extract was recorded at 326 nm wavelength.

Phenolic compounds were identified by comparing their retention times and UV-vis spectra similarity with the standard substances. Each HPLC-grade standard substance was dissolved in ethanol at 100 mg/L concentration, and the calibration curves for the phenolic compounds quantification were obtained by injecting five solutions with concentrations ranging from 0.5–100 mg/L for each standard. For the quantification of each standard phenolic compound, a chromatogram was recorded at the maximum absorption wavelength, and the retention time and coefficient of the calibration curve were presented in Supplementary Information.

### 2.6. Preparation of SE Extract-Loaded Lipid Vesicles

Three types of lipid vesicles, liposomes, transfersomes and ethosomes were employed to entrap the SE extract. All lipid vesicles were prepared using PC as lipid due to its amphiphilic nature (polar head group and lipophilic tail), and its capacity to organize itself spontaneously into bilayers. Apart from PC, cholesterol was included in liposomes formulation due to its influence on the phospholipid alkyl chain free movement, which led to a reduced bilayer permeability and in vivo and in vitro enhanced stability of the liposomes. Sodium cholate was used in transfersomes formulation as “edge activator” due to its ability to destabilize the lipid bilayers, providing more flexibility to the transfersomes.

The SE-loaded liposomes and transfersomes were prepared using thin-film hydration method, followed by ultrasound treatment and extrusion. Briefly, the SE extract, phosphatidylcholine and cholesterol (for liposomes) or sodium cholate (for transfersomes) were dissolved in 5 mL chloroform/methanol mixture (1/3, *v*/*v*) and methanol, respectively. Then, the solutions were kept at room temperature overnight to facilitate the phosphatidylcholine swelling. The lipid solutions were poured into a round-bottom flask and evaporated in a rotary evaporator at 35 °C for 2 h. After solvent removal, the resulting thin lipid film was hydrated with distilled water at 35 °C. The dispersions were kept for 2 h at room temperature for stabilization.

The formulations based on ethosomes were prepared using the cold method. Firstly, phosphatidylcholine was dissolved in ethanol and kept for 24 h at room temperature for swelling. Then, SE extract was added to phosphatidylcholine solution. The resulted mixture was kept under vigorous stirring in a covered round-bottom flask to avoid ethanol evaporation, and then heated distilled water at 30 °C was added slowly under stirring. The dispersions were kept at room temperature for 30 min under continuous magnetic stirring.

All obtained formulations were ultrasounds treated in an ultrasonic bath (Sonorex Digital 10P, Bandelin Electronic GmbH & Co., Berlin, Germany) filled with ice, for 20 min, at a controlled power of 20% of amplitude. The lipid-containing extract vesicles were extruded first using 0.4 μm and then 0.2 μm pore size filters with 5 cycles for each pore size. Empty liposomes, transfersomes and ethosomes were prepared as the controls. All samples were obtained in triplicate and stored in a refrigerator at 8 °C for no more than 48 h prior to further use. More details about lipid formulations containing SE extract are provided in Table 1.

### 2.7. Characterization of SE−Loaded Lipid Vesicles

For the SE extract-loaded lipid vesicles the size, polydispersity index (PDI) and entrapment efficiency (EE), as well as zeta potential values, were determined. The first step for determination of entrapment efficiency was the separation of SE-loaded lipid vesicles from the free extract by centrifugation (10,000 rpm at 5 °C/30 min), and then they were redispersed in water. The centrifugation was repeated twice, and then the sediment was mixed with 0.5 mL Triton X-100 (0.5%) and subsequently vortexed for breaking down the lipid membranes. The final suspension was diluted ten times with methanol. EE was calculated using the following Equation (1):

(1) $$EE\left(\%\right)\;=\;\frac{Q_{1}}{Q_{t}}\;\times \;100$$

here *Q_1_* is the amount of polyphenols entrapped in lipid vesicles and *Q_t_* is the total amount of polyphenols from the extract. The total of the polyphenol amounts entrapped in lipid vesicles was assessed by Folin-Ciocalteu assay, and empty lipid vesicles were used as control.

The particle size and PDI of lipid vesicles were determined by dynamic light scattering (DLS) using a particle size analyzer (Beckman Coulter N4 PCS Submicron, Coulter Company, measurement range of 3 nm–3 µm). The lipid vesicle dispersions were diluted ten times with water, and the measurements were performed at 25 °C in triplicate with ten runs for each determination. The zeta potential values, ξ, of SE-loaded lipid vesicles were determined using a Zetasizer (Malvern Panalytical Ltd., Malvern, UK). The ξ values were measured on undiluted samples at 25 °C in triplicate.

The lipid vesicles containing SE extract were investigated using various microscopy techniques. Atomic force microscopy (AFM) was performed with an XE-100 microscope (Park Systems Corp., Suwon, Korea) in true non-contact^TM^ mode, as recommended for soft sample scanning. The scanner of the XE100 apparatus was equipped with flexure-guided, cross-talk eliminated scanners. All AFM images were recorded with sharp tips (<8 nm tip apex), NCHR type from Nanosensors^TM^ (Neuchâtel, Switzerland), of 125 mm length, 30 mm width, spring constant 42 N/m, and 330 kHz resonance frequency. For the AFM experiments, a small quantity of the sample was dispersed in ultra-pure water (Millipore Direct-Q3UV water system with Biopack UF cartridge), deposited on a clean silicon wafer and dried at room temperature. The recorded AFM images were processed with the XEI program (version 1.8.0, Park Systems) for displaying purposes. Representative line scans are presented (below each image) showing in detail the z-scale of the images and the dimensions of the selected features (particles), as indicated along the selected line between two red arrows.

Dark-field microscopy was performed using a CytoViva system (CytoViva Inc., Auburn, AL, USA). Dark-field microscopy combined with hyperspectral imagery is a novel optical technique that allows for the localization of different types of unmarked particles with dimensions less than the diffraction limit. This is possible because on the hyperspectral camera is collected only the reflected or elastically-scattered light as a unique optical fingerprint of each material. It is based on the oblique illumination of the sample, such that the direct light is not collected, allowing it to distinguish objects with similar refractive indexes as glass coverslips.

Also, SE extract-loaded lipid vesicles were analysed using scanning electron microscopy (SEM) with a Tescan Vega 3 LMH microscope (Brno, Czech). The samples were prepared from a drop of lipid suspension that was further dried in a vacuum. Selected freeze-dried lipid vesicles were also investigated by SEM.

### 2.8. In Vitro Release Polyphenols from SE Loaded Lipid Vesicles

The polyphenols release profiles from lipid vesicle formulations were carried out using a dialysis membrane under sink conditions. The dialysis bag (14,000 molecular weight cut-off from Sigma-Aldrich, USA) in which was put a lipid vesicles suspension (1.0 mL) was immersed into 50 mL 0.1 M phosphate buffer solution of pH 7.4 at 37 °C under magnetic stirring (100 rpm/min). Aliquots of 1.0 mL were withdrawn at predetermined intervals, and the release medium was refilled with the same volume.

The polyphenols from the released medium were determined by UV-vis spectrophotometry using Folin-Ciocalteu assay and compared with the free extract dissolution in the same medium, at a PBS pH of 7.4. The polyphenols release profiles were performed in triplicate.

### 2.9. Determination of Radical Scavenging Activity

The radical scavenging activity of the extract was evaluated using the Sanchez-Moreno et al. assay [36]. Briefly, each sample (50 μL) at different concentrations (serial dilution of SE extract ranging from 0.001–10 mg/mL) was mixed with 2950 μL of DPPH methanolic solution (0.025 g/L). All mixtures were shaken vigorously and kept in dark conditions at room temperature for 30 min. The solution absorbance was measured at 517 nm wavelength. The radical scavenger activity was evaluated based on the percentage of DPPH radical inhibition and computed using Equation (2):

(2) $$\%RSA\;=\;\frac{A_{0}-A_{sample}}{A_{0}}\;\times \;100$$

where *A_0_* is the control absorbance and *A_sample_* is the sample absorbance.

Quercetin (0.0001–1 mg/mL) and caffeic acid (0.0001–1 mg/mL) were used as positive controls. Experiments for radical scavenger capacity assessment were carried out in five replicates.

### 2.10. Induced Oxidative Stress Assay

The L-929 fibroblast cells were seeded on glass coverslips or plastic wells 2 days before experiments to achieve a confluence of ~85% and transferred to 96-well assay plates at a density of 10^5^ cells/mL. Then, the oxidative stress was induced by exposure to various concentrations of hydrogen peroxide aqueous solution (1, 10, 20, 50 and 100 mM) for 4 h for determination of the IC_50_ cell viability. For assessing the cytoprotective effect of SE extract free from and entrapped into lipid vesicles, L-929 cells were treated for 1, 12 and 24 h with empty lipid vesicles, free SE extract and SE-loaded lipid vesicles, and then the cell viability was determined using MTS assay. The medium was changed, and the cells were exposed to the IC_50_ concentration of hydrogen peroxide solution (50 mM) for another 24 h. As a reference substance, ascorbic acid was used in the same concentrations as tested lipid formulations (5, 10 and 25 µg/mL). The absorbance was read using a Microplate Reader (Chameleon V Plate Reader, LKB Instruments, Victoria, Australia) at a wavelength of 490 nm. The control was considered as the culture medium with and without hydrogen peroxide. All samples were sterilized by UV exposure for two hours. Experiments were carried out in triplicate.

### 2.11. Statistical Analysis

Statistical analysis of the data was performed using SPSS 18.0 (SPSS Inc., Chicago, IL, USA). The results were presented as mean value ± SD. Differences were considered significant where *p* < 0.05. The IC_50_ values for RSA and cell viability were computed using GraphPad Prism version 7 software (GraphPad Software Inc., San Diego, CA, USA).

## 3. Results

### 3.1. Characterization of SE Extract

SE leaves were selected as vegetal material from a database containing plants with radical scavenging activity considering its significant amount of phenolic compounds and superior valorisation of vegetal material. The SE leaves extract was characterized for total polyphenols content, chemical profiling, radical scavenging capacity, and cytoprotective effect on hydrogen peroxide-induced toxicity on the L-929 fibroblast cell line.

The hydroalcoholic extract from SE leaves exhibited a significant polyphenols content, 25.50 ± 0.010 mg GAE/g dry material, in accordance with the literature data. For instance, Feizbakhsh et al. (2010) reported a value for phenolic content of 27.40 ± 0.8 mg GAE/g dry material for an ethanolic extract of SE leaves [37].

**Figure 1:**
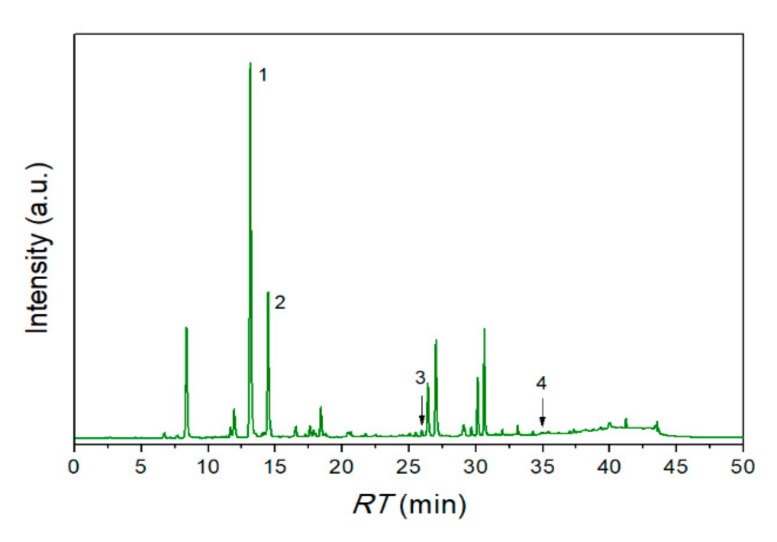
Chromatogram of SE extract at 326 nm: **1**–Chlorogenic acid: *RT* = 13.160 ± 0.002 min; *c* = 14.389 ± 0.018 mg/g extract; **2**–Caffeic acid: *RT* = 14.500 ± 0.004 min; *c* = 2.997 ± 0.004 mg/g extract; **3**–Rutin: *RT* = 25.970 ± 0.002; *c* = 0.564 ± 0.001 mg/g extract; **4**–Quercetin: *RT* = 34.952 ± 0.002, *c* = 0.073 ± 0.001 mg/g extract.

The SE extract chemical profiling was investigated using reverse-phase HPLC analysis, in which was used a mixture of eleven phenolic compounds, from which four were detected and quantified (Figure 1). The most abundant compound was chlorogenic acid (14.389 ± 0.018 mg/g extract). Also, caffeic acid was presented in a significant amount (2.997 ± 0.004 mg/g extract). On the other hand, rutin (0.564 ± 0.001 mg/g extract) and quercetin (0.073 ± 0.001 mg/g extract) were also identified and quantified, but they were in low concentrations in the SE extract. The presence of identified compounds was consistent with previous studies [37].

The radical scavenging capacity of SE extract was assessed using the DPPH method that reflects the capacity of a compound to induce the inhibition of 50% of the DPPH radical (IC_50_). In comparison with the IC_50_ of SE (10.33 ± 0.056 µg/mL), it was approximately 1.7 and 2.4 times lower than that of quercetin (5.97 ± 0.026 μg/mL) and caffeic acid (4.370 ± 0.022 μg/mL), respectively.

### 3.2. Characterization of SE Extract−Loaded Lipid Vesicles

The features of lipid vesicles influence their in vitro and in vivo behaviour; hence, their characterization is an essential step in their application in biomedical and cosmetic field. The size, PDI, EE and stability over three months of SE extract-loaded lipid vesicles were determined using empty lipid vesicles as control. The composition of lipid vesicles and their features were gathered in Table 1.

The formulations were selected after one-factor-at-a-time experiment (see Supplementary Information). Four factors (PC/SE extract ratio, evaporation temperature, stirring rate, PC/cholesterol (sodium cholate) ratio) were assessed for liposomes and transfersomes, respectively. Two factors (PC/SE extract ratio and water/ethanol ratio) were assessed for ethosomes.

The size enlargement after the extract loading process was caused by the incorporation of phytochemicals in the vesicle structure. The SE extract contains phytocompounds with different polarity. Therefore, according to the literature [38] their localization within lipid vesicles is different, for instance, non-polar compounds are located in the bilayer, and the polar ones in the aqueous inner core, resulting an increase of particle size. Also, the incorporation of non-polar compounds probably induces a fluidization effect on lipid vesicles membrane by producing a PC perturbation leading to a deep insertion of polyphenols in phospholipid bilayer, as reported for eugenol and isoeugenol [39]. The results are in agreement with literature data, an increase of particle size being reported because of incorporation of grape-seed [40,41], liquorice [38] and *Polygonum aviculare* [42] extracts. The size of SE-loaded lipid vesicles increases in order: liposomes < transfersomes < ethosomes. Liposomes and tranfersomes had lower size due to the presence of cholesterol, and sodium cholate in the lipid bilayer that compete with the extract. So, the bilayer could accommodate only a low amount of polyphenols, leading to a decreased size.

The PDI values show the homogeneity of systems. Generally, PDI values less than 0.1 indicate a homogeneous population, while values higher than 0.3 show a big heterogeneity [43]. All loaded lipid vesicles showed narrow size distribution (PDI < 0.21), proving a good homogeneity and a low tendency of aggregation. The incorporation of SE extract improved the homogeneity, while the empty vesicles had a wider size distribution than SE-loaded vesicles in agreement with literature data [44,45]. The zeta potential values were in the range of −43 ± 1.03 mV ÷ −37 ± 0.23 mV being in accordance with the literature data, all samples indicated moderate stability [46].

**Figure 2:**
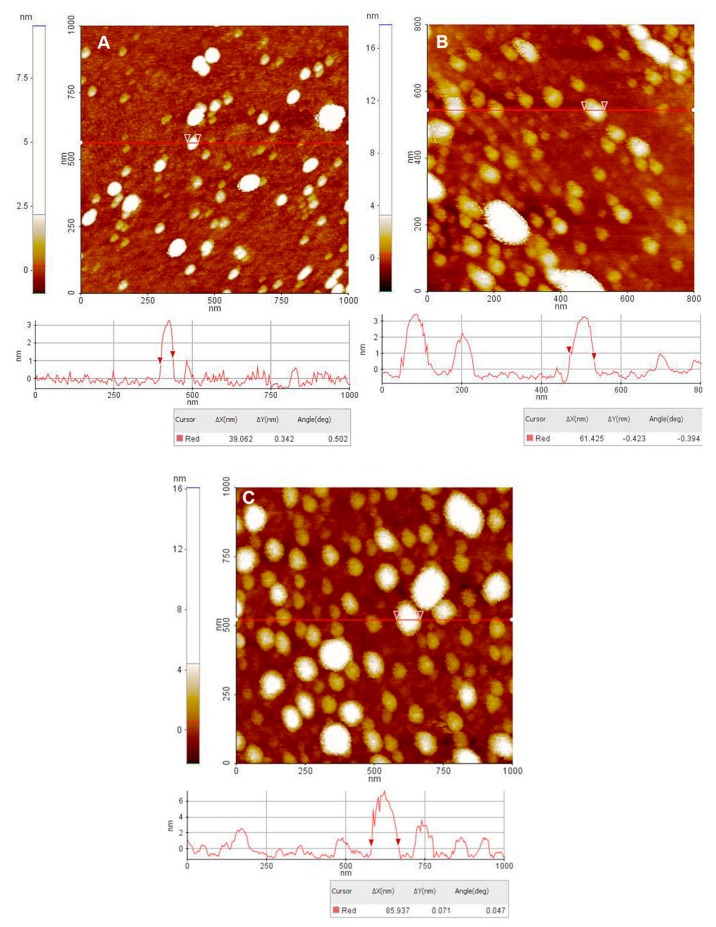
2D AFM images together with representative line scans (surface profile) of the imaged samples: (**A**) liposomes, (**B**) transferosomes and (**C**) ethosomes.

The AFM investigation of SE-loaded samples revealed randomly distributed quasi-spherical particles for all type of lipid vesicles, with size of tens of nm (selected vesicles having the average diameter of 39 nm in the case of liposomes (Figure 2A), 62 nm for transfersomes (Figure 2B) and 86 nm for ethosomes (Figure 2C)). Larger and more homogeneously distributed lipid vesicles are noticed for SE@E sample (Figure 2C), while smaller and more compacted for SE@L sample (Figure 2A). The vesicular nature of samples is confirmed by their spiky (noisy-like) profile (see for examples the lines below Figure 2B,C).

The AFM images of SE extract-loaded lipid vesicles showed particles with lower dimensions than in the case of SEM analysis (see Supplementary Information, Figures S4 and S5), probably because of the aggregation of nanoparticles during the freeze-drying process. However, the aggregates of liposomes preserve the shape of primary nanoparticles, having uniform size, in the range of 1–3 μm. The size of lipid vesicles obtained by AFM analysis are smaller than the hydrodynamic diameters measured by DLS or the size determined by SEM (Supplementary Information, Figure S6), but the same order of increase for lipid vesicle dimensions was noticed (Table 1).

**Figure 3:**
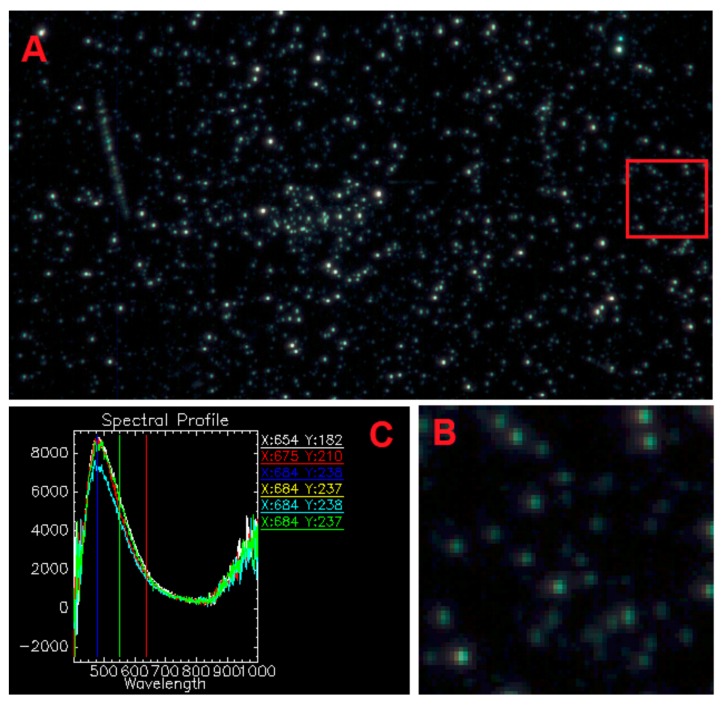
Image recorded by enhanced dark-field microscopy using Cytoviva (**A**); magnification of marked area from figure A corrected with the lamp spectrum (**B**) and spectra collected in the isolated points (**C**).

Hyperspectral images of SE@E sample were obtained using enhanced dark field microscopy mounted on an Olympus BX51 upright microscope (Olympus, Tokyo, Japan) equipped with a CytoViva^®^ enhanced dark-field condenser (CytoViva Inc., Auburn, AL, USA) and patented illumination with liquid-core optical fiber. Using a 60× microscope objective oil immersed, the sample was scanned (the exposure time for a frame is established in accordance with the producer procedure and it is of the order of few seconds). The acquired images are corrected with the lamp spectrum (Figure 3B) and spectra are collected in the isolated points and cumulated on the same plot (Figure 3C). It is observed that points where nanoparticles are located are isolated from each other, and the light scattering spectra show similar maximum intensities, indicating a narrow size distribution of lipid vesicles.

The entrapment efficiency of SE extract in all lipid vesicles was above 75% and increased in order liposomes < transfersomes < ethosomes, the applied preparation methods being efficient. Similar results were reported for the entrapment efficiency of *Polygonum aviculare* [42], *Glycyrrhiza glabra* L. [38] and *Artemisia arborescens* [47]. For liposomes it was obtained the lowest EE because of the cholesterol presence that lowered the incorporation efficiency of polyphenols. The influence of cholesterol can be explained by two phenomena: (*i*) lipophilic polyphenols compete with cholesterol molecules for the lipophilic space in the lipid bilayer [48] and/or (*ii*) cholesterol decreases the flexibility of the lipid bilayer and thus, the possibility of integration of lipophilic molecules into the lipid membrane [49].

On the other hand, sodium cholate used as “edge activator” in transfersomes may improve the flexibility of lipid bilayer, leading to the accommodation of a higher amount of polyphenols than in liposomes. Moreover, transfersomes had a smaller quantity of extract than ethosomes, their EE being slightly hindered by the competition between SE extract and the “edge activator” for the bilayer space in agreement with Gupta et al. results [50].

For the stability of loaded lipid vesicles evaluation, the samples were kept in a 10 mL amber-coloured glass vial and stored at 4 °C for 3 months. Since all formulations were protected by light, the oxidation and hydrolysis of the lipids caused by light were avoided. To assess the stability of samples, EE parameter was evaluated after different storage periods of time (0, 1, 2, 3 months). The SE-loaded lipid vesicles were stable for at least three months, with approximately the same amount of phytocompounds entrapped after one month (extract compounds loss < 0.65%) and after 3 months (extract compounds loss < 3.35%). No sedimentation of SE-loaded lipid vesicles was observed during storage, probably because the brownian motion and the diffusion rate, which in the case of nano-sized formulations are higher than the gravitational-induced sedimentation rate.

### 3.3. In Vitro Polyphenols Release Study

**Figure 4:**
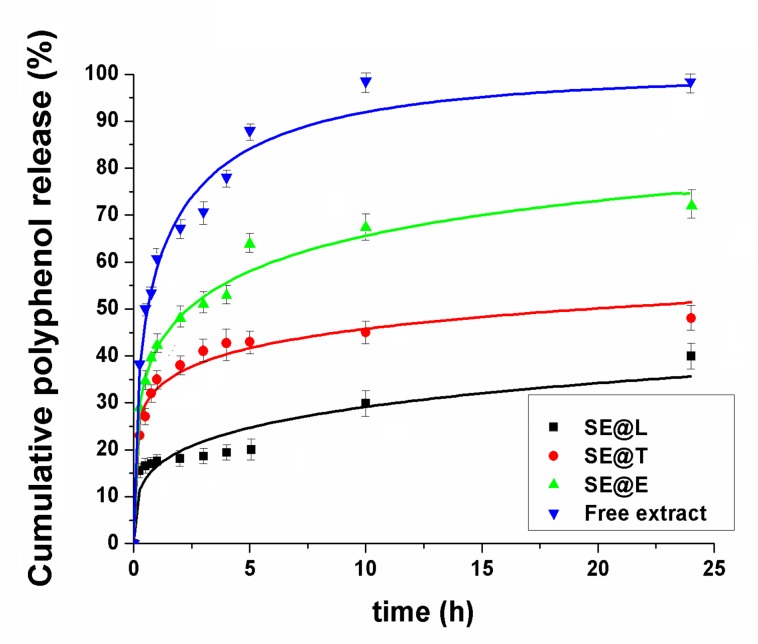
Polyphenols release profiles from SE extract-loaded lipid vesicles in comparison with free SE extract solubilization in PBS of pH 7.4 fitted with the Weibull model.

The in vitro release of polyphenols from lipid vesicles was determined in PBS pH 7.4 at 37 °C, and the experimental data were presented in Figure 4. The amount of polyphenols delivered from SE extract was assessed using a Folin-Ciocalteu assay. One can notice that the release of polyphenols from the free extract exhibited a “burst effect”, while in the case of SE extract entrapped into lipid vesicles, lower kinetics were determined. After 10 h, almost all polyphenols from the free extract were released (98.51 ± 1.07%), while for all lipid formulations, the release rate was lower (bellow 70%). The liposome-based formulations showed the slowest polyphenols release rate due to the presence of cholesterol, which increased the rigidity and decreased the permeability of the lipid bilayer. Transfersomes and ethosomes, because of their ultra-deformable nature, determined faster polyphenols release kinetics. To evaluate the mechanism of polyphenols release, three kinetics models were applied: Weibull (Equation (3)), Korsmeyer-Peppas (Equation (4)) and Higuchi (Equation (5)):

(3) $$\frac{M\left(t\right)}{M\left(\infty \right)}\;=\;1\;-\;e^{-at^{b}}$$

(4) $$\frac{M\left(t\right)}{M\left(\infty \right)}\;=\;k_{KP}\times \;t^{n}$$

(5) $$\frac{M\left(t\right)}{M\left(\infty \right)}\;=\;k_{H}\times \;t^{1/2}$$

where *M(t)* represents polyphenols released at time *t* and *M(∞)* represents the total amount of polyphenol located in the carrier; *k_KP_*, *k_H_* are Korsmeyer-Peppas and Higuchi constants, and *a*, *b* are Weibull function parameters.

**Figure 5:**
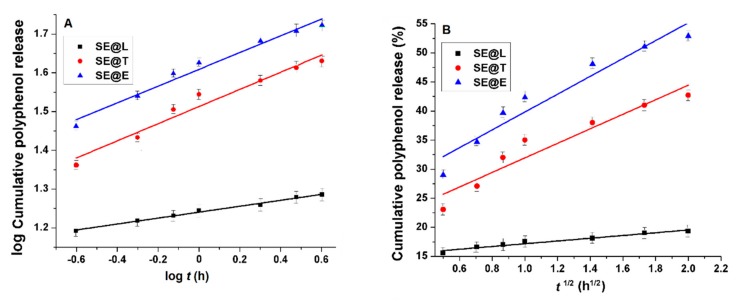
Polyphenols release profiles from SE extract-loaded lipid vesicles fitted with the Korsmayer-Peppas (**A**) and Higuchi (**B**) models.

In Table 2 are listed the correlation coefficients and parameters of mathematical models used for fitting the experimental data of polyphenol release from lipid vesicles in comparison with phytochemicals solubilization in PBS from SE hydro-alcoholic extract. The highest correlation coefficients were obtained for the Weibull Equation, for which *a* parameter is a measure of the bio-compounds delivery rate and parameter *b* indicates their transport [51]. Values of *b* parameter lower than 0.75 suggest a Fickian diffusion of biomolecules, while for higher values of *b* indicate a non-Fickian transport [52]. From all lipid-type formulations, *b* < 0.75 suggests a Fickian diffusion. Korsmeyer-Peppas and Higuchi models were applied only for the first 60% cumulative release of polyphenols with correlation coefficients higher than 0.96 and 0.92, respectively (Figure 5). In the Korsmeyer-Peppas’s model, the *k_KP_* is a constant that depends on the carrier characteristics, and the coefficient *n* shows the nature of the release mechanism. When *n* ≤ 0.45, the release is dominated by the Fickian diffusion mechanism; the 0.45 < *n* < 0.89 release follows anomalous diffusion (non-Fickian diffusion), and *n* > 0.89 follows a super-case-II transport mechanism [53]. The *n* and *k_KP_* values are listed in Table 2. For all samples, the coefficient *n* is lower than 0.45, indicating a Fickian diffusion mechanism in agreement with the Weibull model. In the Higuchi’s model, *k_H_* is a constant proportional with the release rate in the burst stage of the delivery process. The SE-loaded liposomes presented a lower value for *k*_H_ (Table 2) than the other lipid formulations, indicating a less intense burst effect, probably due to their cholesterol content, which increased the rigidity of the lipid bilayer and altered its permeability. Also, Gibis et al. reported a lower burst effect of polyphenols from grape seed extract-loaded liposomes than their solubilization process, with the polyphenols transport having a diffusion-controlled mechanism [54,55].

### 3.4. Effect of Pre−Treatment with SE Extract Free and Loaded into Lipid Vesicles against H_2_O_2_−Induced Toxicity on L−929 Fibroblast Cell Line

**Figure 6:**
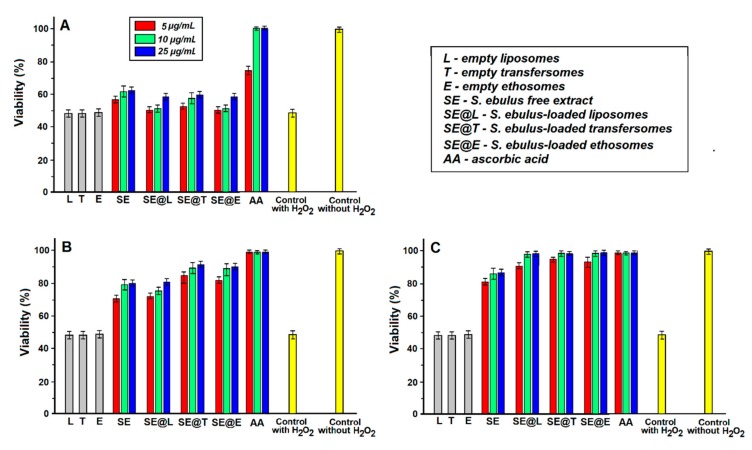
Effect of H_2_O_2_ on L-929 cell viability after exposure. Viability of L-929 mouse fibroblast cells after 1 h (**A**), 12 h (**B**) and 24 h (**C**) pre-treatment with free and loaded-SE leaves extract before exposure at hydrogen peroxide solution of 50 mM concentration during 4 h are shown.

The viability of L-929 mouse fibroblast cells was evaluated after 4 h exposure at different concentrations of hydrogen peroxide aqueous solution in the range of 1–100 mM, its cytotoxicity being dose-dependent. The IC_50_ concentration of H_2_O_2_ was 50 mM. Hydrogen peroxide is not very reactive, but sometimes it can be toxic because it produces hydroxyl radicals [56]. Adding hydrogen peroxide solution to L-929 cells caused a significant increase in reactive oxygen species (ROS) level, which probably caused the death of L-929 cells. Three concentrations (5, 10, 25 μg/mL) for SE extract and SE-loaded lipid vesicles were selected, which are non-toxic and could prevent H_2_O_2_-induced cytotoxicity. Hence, L-929 fibroblast cells were pre-treated with free SE extract and SE-loaded lipid vesicles before adding hydrogen peroxide solution with IC_50_ concentration. One can notice enhanced viability when cells were previously treated with free and loaded SE extract (Figure 6), which suggested a cytoprotective effect against ROS of all free and loaded SE extract into lipid vesicles.

Fibroblast cells developed some protective effects against the oxidative stress that was illustrated by a high cells’ viability when a long pre-treatment (24 h) with SE extract free and loaded into lipid vesicles was applied. However, the effect was not significant in the case of short-term exposure of cells at SE extract free and loaded (1 h pre-treatment), suggesting that the development of the cytoprotective effect requires a long intracellular presence of the SE extract. All samples were able to protect L-929 cells against H_2_O_2_-induced cytotoxicity, probably due to their polyphenol content. Many studies confirmed that polyphenols may prevent the degenerative effects caused by oxidative stress, which could determine various deleterious events [57]. Polyphenols could act as antioxidants [58], and the mechanism has already been studied in vivo [59]. The main effect of phenolic compounds as antioxidants was determined by the presence of characteristic moieties, mainly phenylhydroxyl groups that can act as hydrogen donors, and hence can react with ROS and RNS (reactive nitrogen species) forming phenoxy radicals [56]. Another interesting mechanism for the radical scavenging activity of polyphenols was revealed by Rise-Evans et al., which showed that phenolic acids can stabilize or relocate unpaired electrons, chelate transition metal ions, catalyse free radicals (oxidation reactions), and may act as oxidation inhibitors [60].

These in vitro experiments demonstrated that polyphenols from SE leaves extract have radical scavenging capacity resulting in a cytoprotective effect even at a low concentration of 5 ug/mL. It is known that the use of lipid vesicles as carriers could help cell internalization through endocytosis of polyphenols [61]. Hence, the SE extract loaded into lipid vesicles could be considered a good method to improve polyphenol cell internalization.

## 4. Conclusions

A hydroalcoholic polyphenols extract from *Sambucus ebulus* leaves was prepared and characterized using total polyphenols content (25.50 ± 0.010 mg GAE/g dry material), and radical scavenging activity using DPPH assay (IC_50_ = 10.33 ± 0.056 µg/mL) and reverse phase HPLC-PDA analysis, which showed the presence of chlorogenic acid (in the highest amount among the identified compounds), caffeic acid, rutin, and quercetin.

SE leaves extract was further loaded into three types of lipid vesicles: liposomes, transfersomes and ethosomes, which were characterized through their entrapment efficiency, AFM, enhanced dark-field microscopy, SEM and DLS measurements. All prepared SE-loaded lipid vesicles showed good entrapment efficiency (in the range of 75–85%) and good stability over three months when stored at 4 °C. The microscopy investigation proved the nanosized lipid vesicles with a narrow size distribution, the smallest being liposomes that are more hydrophobic and less deformable than transfersomes and ethosomes, while the largest ones were ethosomes, which exhibited the highest entrapment efficiency.

Polyphenols from SE leaves extract had slower release kinetics when were loaded in lipid vesicles in PBS pH 7.4 than their solubilisation in the same environment.

It was shown a cytoprotective effect of *Sambucus ebulus* leaves extract free and loaded into lipid vesicles against hydrogen peroxide induced-toxicity on L-929 mouse fibroblasts cell line. However, a short treatment of only 1 h with SE extract did not exhibit a significant cytoprotective action on cells, it was required at least 12 h of pre-treatment before exposure to hydrogen peroxide solution.

These results suggest that lipid vesicles could be exploited as carriers for polyphenols for biomedical or cosmetic applications, but further investigations are needed.

## Figures and Tables

**Table 1:** The characteristics of *Sambucus ebulus* (SE) extract-loaded lipid vesicles.

Sample	Formulation	EE (%)	Size (nm)	PDI	ξ (mV)
SE@L	PC:Cholesterol:SE = 10:1:2 (*w*/*w*)	80.05 ± 0.51	123 ± 2.50	0.182 ± 0.01	−43 ± 1.03
SE@T	PC:Sodium cholate:SE = 8:2:2 (*w*/*w*)	75.10 ± 1.12	155 ± 3.31	0.161 ± 0.02	−39 ± 0.50
SE@E	PC:SE = 8:2.5 (*w*/*w*); EtOH:H_2_O = 7:3 (*v*/*v*)	85.10 ± 1.50	190 ± 2.53	0.209 ± 0.01	−37 ± 0.23
L	PC:Cholesterol = 10:1 (*w*/*w*)	-	49 ± 1.52	0.440 ± 0.01	-
T	PC:Sodium cholate = 8:2 (*w*/*w*)	-	105 ± 0.23	0.379 ± 0.02	-

**Table 2:** Correlation coefficients and parameters of the fitted experimental data.

Sample	Weibull			Korsmayer-Peppas			Higuchi	
	*a*	*b*	*R* ^2^	*n*	*k_KP_*	*R* ^2^	*k_H_*	*R* ^2^
Free extract	0.885	0.455	0.918	-	-	-	-	-
SE@L	0.181	0.280	0.983	0.076	17.390	0.991	2.363	0.973
SE@T	0.400	0.184	0.984	0.221	32.568	0.963	12.507	0.922
SE@E	0.538	0.297	0.970	0.216	40.640	0.978	16.980	0.949

## Supplementary Materials

The following are available online at https://www.mdpi.com/2079-4991/10/1/56/s1, Figure S1: Influence of a single factor on entrapment efficiency of SE extract-loaded liposomes: PC/cholesterol ratio (A), evaporation temperature (B), stirring rate (C), SE extract amount (D); Figure S2: Influence of a single factor on entrapment efficiency of SE extract-loaded transfersomes: PC/sodium cholate ratio (A), evaporation temperature (B), stirring rate (C), SE extract amount (D); Figure S3: Influence of a single factor on entrapment efficiency of SE extract-loaded ethosomes: PC/SE extract ratio (A), water/ethanol ratio (B); Figure S4: SEM image of freeze dried SE extract-loaded liposomes; Figure S5: Enhanced color view 2D AFM of SE extract-loaded lipid vesicles: liposomes (A), transfersomes (B) and ethosomes (C); Figure S6: SEM images of SE extract loaded lipid vesicles dried in vacuum; Table S1: Standard HPLC phenolic compounds: retention time (*RT*) and standard deviation (SD), maximum wavelength (*λ**max*), calibration curve *y* = *ax* + *b* and correlation coefficients (*R^2^*).
