# Supplementary material for: Polyphenolic Extract from Sambucus ebulus L. Leaves Free and Loaded into Lipid Vesicles
Source: Nanomaterials (Basel). 2019 Dec 25;10(1):56. doi: 10.3390/nano10010056 (PMC7023427; doi:10.3390/nano10010056)
Supplement: Supplementary file 1 [file nanomaterials-10-00056-s001.pdf]

Supplmentary Materials: Polyphenolic Extract from *Sambucus ebulus* L. Leaves Free and Loaded into Lipid Vesicles

Ramona-Daniela Păvăloiu [1,2], Fawzia Sha'at [1,2], Corina Bubueanu [1], Mihaela Deaconu [2], Georgeta Neagu [1], Mousa Sha'at [3], Mihai Anastasescu [4], Mona Mihailescu [5], Cristian Matei [2], Gheorghe Nechifor [2] and Daniela Berger [2,*]

[1] National Institute for Chemical-Pharmaceutical Research and Development-ICCF Bucharest, Vitan Avenue 112, 031299 Bucharest, Romania; pavaloiu_daniella@yahoo.com (R.-D.P).; fawzya.shaat@gmail.com (F.S.); corina.bubueanu@yahoo.com (C.B.); getabios@yahoo.com (G.N.)
[2] Faculty of Applied Chemistry and Materials Science, University "Politehnica" of Bucharest, 1–7 Gheorghe Polizu St., 011061 Bucharest, Romania; mihaela_deaconu@yahoo.com (M.D.); cristian.matei@upb.ro (C.M.); doru.nechifor@yahoo.com (G.N.)
[3] Faculty of Pharmacy, University of Medicine and Pharmacy Grigore T. Popa, Iasi, Universitatii Avenue, 16, 700115 Iasi, Romania; mousa.shaat1@gmail.com
[4] "Ilie Murgulescu" Institute of Physical-Chemistry, Romanian Academy, Splaiul Independentei no. 202, 060021 Bucharest, Romania; manastasescu@icf.ro
[5] Physics Department, University "Politehnica" of Bucharest, Splaiul Independetei no 313, 060042 Bucharest, Romania; mona.mihailescu@yahoo.com
* Correspondence: daniela.berger@upb.ro

1. HPLC analysis of *Sambucus ebulus L.* leaves (SE) extract

Table S1. Standard HPLC phenolic compounds: retention time (*RT*) and standard deviation (SD), maximum wavelength (*λmax*), calibration curve *y=ax+b* and correlation coefficients (*R2*)

Compound	*RT* ±SD, min	λ_{max}, nm	*a*	*b*	R_2
Gallic acid	3.59 ± 0.10	271	8371.14	−1207.1	0.9993
Catechin hydrate	12.60 ± 0.27	279	1704.55	−1485.0	0.9974
Chlorogenic acid	13.21 ± 0.26	326	7634.01	−1686.4	0.9984
Caffeic acid	15.24 ± 0.22	323	13188.1	−1501.4	0.9989
p-Coumaric acid	21.82 ± 0.24	309	18220.6	−1642.1	0.9946
Rutin hydrate	25.97 ± 0.02	355	3813.0	−838.9	0.9985
Myricetin	31.76 ± 0.13	373	9150.3	−1464.4	0.9991
Rosmarinic acid	32.24 ± 0.10	330	7282.3	−633.3	0.9988
trans-Resveratrol	33.22 ± 0.11	307	17601.1	−2585.3	0.9989
Quercetin	34.98 ± 0.05	371	9898.8	−723.2	0.9994
Kaempferol	36.08 ± 0.03	367	10549.7	−1296.0	0.9981

2. Establishing the preparation conditions for SE extract-loaded liposomes and transfersomes by using one-factor-at-a-time experiment

Influence of four factors (Phosphatidylcholine from egg yolk (PC)/cholesterol (sodium cholate) ratio, evaporation temperature, stirring rate, SE extract amount) on the entrapment efficiency was assessed for liposomes and transfersomes.

Influence of PC/cholesterol (sodium cholate) ratio on the entrapment efficiency

For PC/cholesterol (sodium cholate) ratio was tested the following values: 10/1, 9/1, 8/2 and 7/3, while the other preparation conditions of liposomes and transfersomes were kept constant (evaporation temperature 35 °C, stirring rate 200 rpm, 10 mg of SE extract). The best results were

obtained for 10/1 PC/cholesterol ratio for liposomes and 8/2 PC/sodium cholate ratio for transfersomes (Figure S1A and S2A). These values were used in further experiments.

Influence of evaporation temperature on the entrapment efficiency

The evaporation temperature was varied in the range of 25–40 °C, and the other preparation conditions of liposomes and transfersomes were kept constant (PC/cholesterol (sodium cholate) ratio 10/1 (8/2), stirring rate 200 rpm, 10 mg of SE extract). One can notice the highest entrapment efficiency for the formulations prepared at 35 °C (Figure S1B and S2B).

Influence of stirring rate on the entrapment efficiency

The tested stirring rate values were 100 rpm, 150 rpm, 200 rpm and 250 rpm, the other preparation conditions being kept constant (PC/cholesterol (sodium cholate) ratio 10/1 (8/2), evaporation temperature 35 °C, 10 mg of SE extract). A stirring rate of 200 rpm determined the best results for both lipid vesicles (Figure S1C).

Influence of SE extract amount on the entrapment efficiency

In order to obtain the best entrapment efficiency, different SE extract amounts were tested: 10 mg, 15 mg, 20 mg, 25 mg, 50 mg, the other preparation conditions of liposomes and transfersomes being kept constant (PC/cholesterol (sodium cholate) ratio 10/1 (8/2), 200 rpm stirring rate, 35 °C evaporation temperature). The results were obtained for 20 mg of SE extract for both lipid vesicles (Figures S1D and S2D).

3. Establishing the preparation conditions of SE extract-loaded ethosomes by using one-factor-at-a-time experiment

Influence of two factors (PC/SE extract ratio and water/ethanol ratio) on the entrapment efficiency was assessed for SE extract-loaded ethosomes.

The tested PC/SE extract ratios were to 8/1, 8/1.5, 8/2, 8/2.5 and 8/3, while the other preparation conditions of ethosomes were kept constant. The PC/SE extract ratio of 8/2.5 showed the best entrapment efficiency (Figure S3A).

Influence of water/ethanol ratio on the entrapment efficiency

The tested following water/ethanol ratios were to 9/1, 8/2, 7/3, 6/4 and 5/5, while the other preparation conditions of ethosomes were kept constant. The best entrapment efficiency was obtained for 7/3 water/ethanol ratio (Figure S3B).

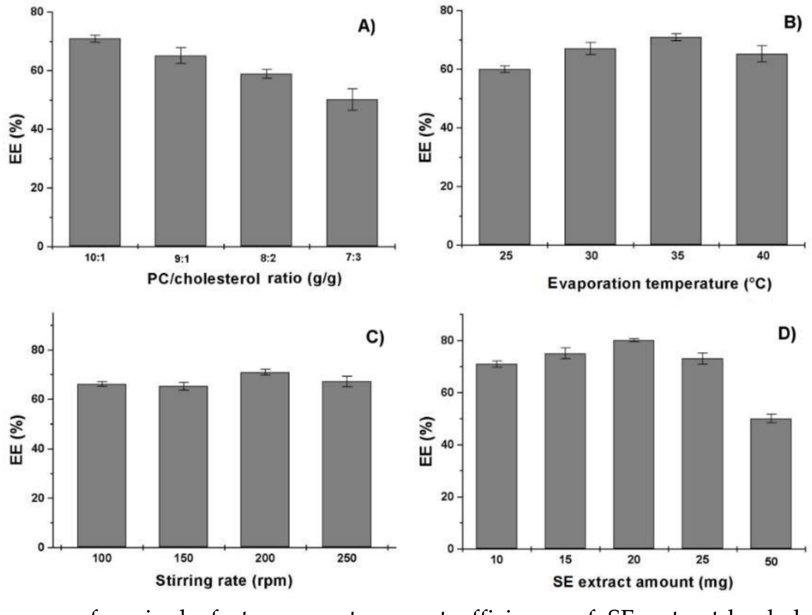

Figure S1. Influence of a single factor on entrapment efficiency of SE extract-loaded liposomes: PC/cholesterol ratio (**A**); evaporation temperature (**B**); stirring rate (**C**); SE extract amount (**D**).

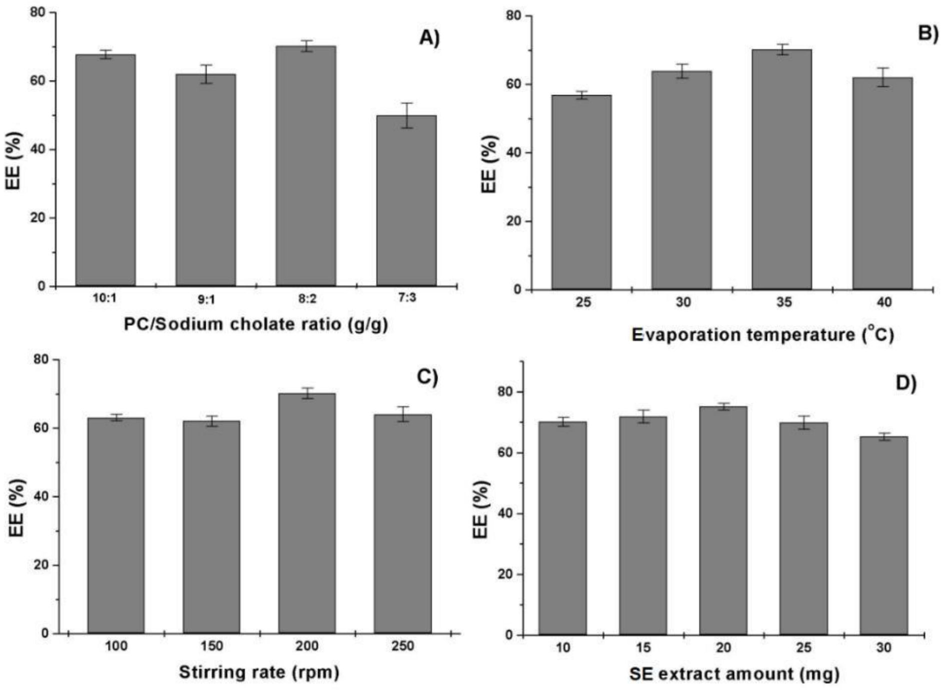

Figure S2. Influence of a single factor on entrapment efficiency of SE extract-loaded transfersomes: PC/sodium cholate ratio (**A**); evaporation temperature (**B**); stirring rate (**C**); SE extract amount (**D**).

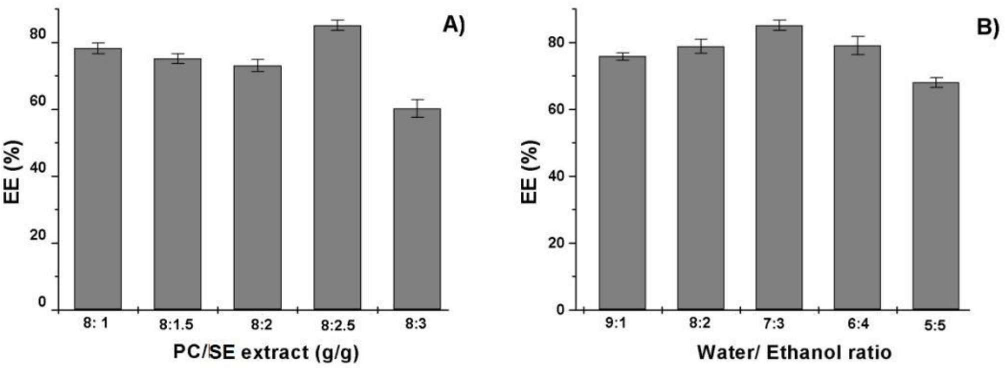

Figure S3. Influence of a single factor on entrapment efficiency of SE extract-loaded ethosomes: PC/SE extract ratio (**A**); water/ethanol ratio (**B**).

4. Characterization of SE extract-loaded lipid vesicles

Freeze dried SE extract-loaded liposomes were characterized by scanning electron microscopy (SEM). The SEM image (Figure S4) showed the uniform size of SE extract loaded liposomes in accordance with DLS analysis.

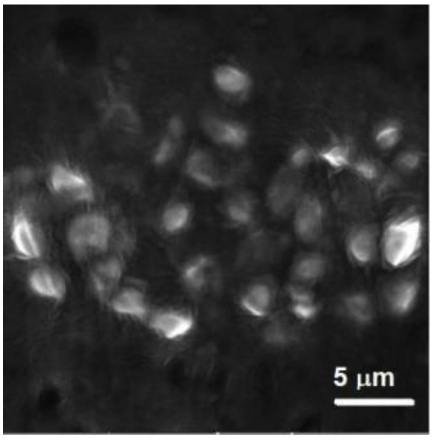

Figure S4. SEM image of SE extract-loaded liposomes

The AFM investigation of SE-loaded lipid vesicles performed on a drop of lipid vesicles suspension in water, deposited on clean Si and dried at room temperature, showed nanosized quasi spherical particles (Figure S5) with lower dimensions than in the case of SEM analysis performed on freeze-dried samples. The aggregates of liposomes containing SE extract preserve the quasi spherical shape of nanoparticles, ranging in 1-3 m. Also, in the AFM images can be observed the core-shell structure of samples (Figure S5).

It was also recorded SEM images on SE extract loaded samples prepared from a drop of lipid suspension, which was further dried in vacuum. As it can be observed in figure S6 that the lipid vesicles are not agglomerated having slightly larger diameters than that measured on AFM analysis, probably because of lipid vesicles flattening. The size of SE extract loaded lipid vesicles increase in the following order: liposomes < trasfersomes < ethosomes.

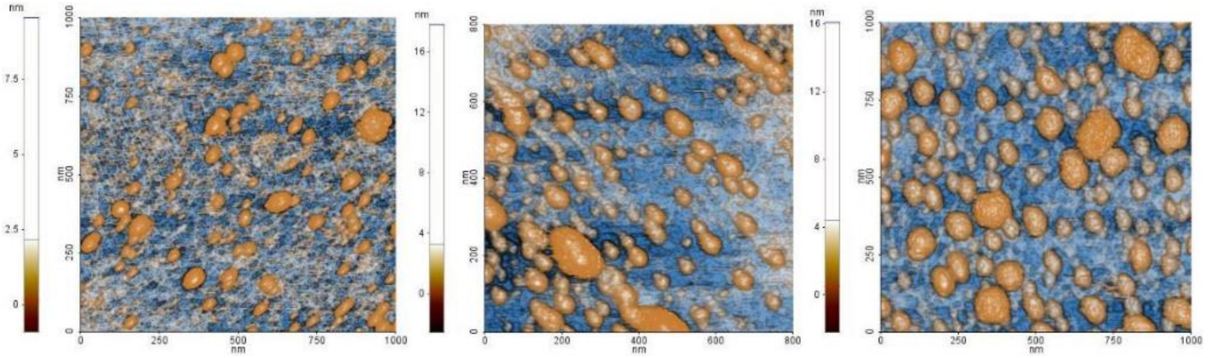

(A) (B) (C)

Figure S5. Enhanced color view 2D AFM of SE extract-loaded lipid vesicles: liposomes (**A**); transfersomes (**B**) and ethosomes (**C**)

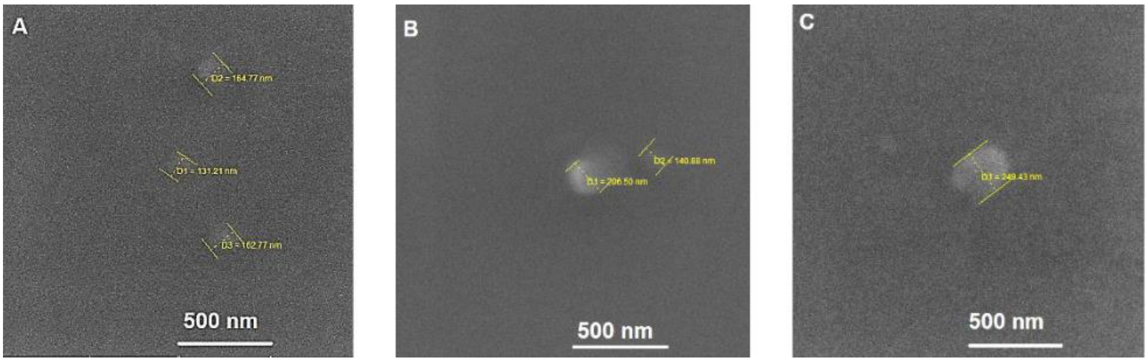

Figure S6. SEM images of SE extract loaded lipid vesicles dried in vacuum
